# The impact of endometrioma on in vitro fertilisation/intra-cytoplasmic injection IVF/ICSI reproductive outcomes: a systematic review and meta-analysis

**DOI:** 10.1007/s00404-020-05796-9

**Published:** 2020-09-26

**Authors:** Sallwa M. Alshehre, Brenda F. Narice, Mark A. Fenwick, Mostafa Metwally

**Affiliations:** 1 Academic Unit of Reproductive and Developmental Medicine, Department of Oncology and Metabolism, University of Sheffield, Tree Root Walk, Sheffield, S10 3HY UK; 2 Academic Unit of Reproductive and Developmental Medicine, Oncology and Metabolism, University of Sheffield, Tree Root Walk, Sheffield, S10 3HY UK; 3 Laboratory Medicine Department, College of Applied Medical Sciences, Umm Al Qura University, Makkah, Saudi Arabia

**Keywords:** Endometrioma, IVF/ICSI, Reproductive outcomes, Oocyte, Fertility

## Abstract

**Background:**

Assisted reproductive technologies (ART) such as in vitro fertilisation (IVF) and intra-cytoplasmic sperm injection (ICSI) are often used to aid fertility in women with endometrioma; however, the implications of endometrioma on ART are unresolved.

**Objective:**

To determine the effect of endometrioma on reproductive outcomes in women undergoing IVF or ICSI.

**Methods:**

A systematic review and meta-analysis was conducted to identify articles examining women who had endometrioma and had undergone IVF or ICSI. Electronic searches were performed in PubMed, BIOSIS and MEDLINE up to September 2019. The primary outcome was live birth rate (LBR). Secondary outcomes included clinical pregnancy rate (CPR), implantation rate (IR), number of oocytes retrieved, number of metaphase II (MII) oocytes retrieved, number of embryos and top-quality embryos and the duration of gonadotrophin stimulation and dose.

**Results:**

Eight studies were included. Where significant heterogeneity between studies was identified, a random-effects model was used. The number of oocytes (weighted means difference; WMD-2.25; 95% CI 3.43 to − 1.06, *p* = 0.0002) and the number of MII oocytes retrieved (WMD-4.64; 95% CI 5.65 to − 3.63, *p* < 0.00001) were significantly lower in women with endometrioma versus controls. All other outcomes, including gonadotrophin dose and duration, the total number of embryos, high-quality embryos, CPR, IR and LBR were similar in women with and without endometrioma.

**Conclusion:**

Even though women with endometriomas had a reduced number of oocytes and MII oocytes retrieved when compared to women without, no other differences in reproductive outcomes were identified. This implies that IVF/ICSI is a beneficial ART approach for women with endometrioma.

## Introduction

Endometriosis is a chronic oestrogen-dependent inflammatory disease, characterised by a histological presence of benign functional endometrial glands or stroma outside the uterine cavity [1, 2]. It is considered the most common benign, but potentially metastatic, gynaecological condition that affects about 7–10% of females of reproductive age in the general population, and it is considered as the main cause of chronic pelvic pain (CPP) [3, 4]. Approximately, 25–40% of infertile women have endometriosis; furthermore, approximately 25% of patients undergoing IVF treatment suffer from endometriosis [5, 6].

An ovarian endometrioma is a growth of ectopic endometrial tissue within the ovary [7] and may appear as a result of metaplasia of the coelomic epithelium or invagination of the ovarian cortex [8, 9]. Approximately, 17–44% of women with endometriosis also have an endometrioma [10, 11]. The definitive cause-and-effect association between the presence of an endometrioma and ovarian function is yet to be clearly established. Some studies have revealed that endometrioma could have a detrimental effect on ovarian function due to the anatomical proximity of the ovarian cyst to the nearby follicular pool—leading to a reduction in the quality and quantity of developing follicles [12, 13]. Other studies have reported that the local inflammation and the toxic content that diffuse from the endometrioma cyst wall to the nearby ovarian tissue may lead to a reduction in the number of oocytes and the quality of the embryos [14–17].

Assisted reproductive technologies (ART), especially in vitro fertilisation (IVF) and intra-cytoplasmic sperm injection (ICSI), are commonly applied to aid sub-fertile and infertile women to conceive, and have shown the highest success rates in treatment strategies for endometriosis-related infertility [18]. However, the influence of endometrioma on reproductive outcomes is still an unresolved issue. Some studies have reported that endometrioma negatively affects the number of oocytes retrieved [19], the quality of embryos [20] and implantation rate [19]. By comparison, others have shown that women with ovarian endometriomas have similar live birth rates compared to control groups, despite fewer oocytes retrieved during IVF treatment [20, 21]. Previous meta-analyses have yielded contradictory results, and have only focused on the effect of the surgical removal of endometrioma on the ART outcomes rather than the effect of the endometrioma itself [16, 22] or included a single-arm group without a control group for comparison [23].

As yet, there is no robust data to identify the exact influence of endometrioma without the intervention of surgery on women undergoing IVF or ICSI. For these reasons, a systematic review and meta-analysis was conducted to determine the influence of endometrioma on reproductive outcomes in women who opt for conservative management.

## Methods

### Outcome measures

The primary outcome was live birth rate; the secondary outcomes were clinical pregnancy rate, mean number of oocytes retrieved, number of metaphase II oocytes retrieved, number of embryos and high-quality embryos, implantation rate, duration of gonadotrophin stimulation and gonadotrophin dose.

### Search strategy and eligibility criteria

The meta-analysis was conducted in accordance with the Preferred Reporting Items for Systematic Reviews and Meta-Analyses (PRISMA) guidelines [24]. A systematic search of electronic databases was conducted in PubMed and Web of Science (BIOSIS, MEDLINE) from inception to September 2019 to obtain the studies focusing on the association between endometrioma and reproductive outcomes. The following combination of relevant search terms was used: endometrioma, endometriosis, ovarian endometrioma, endometriotic ovarian cyst, in vitro fertilisation, intra-cytoplasmic sperm injection, assisted reproductive technologies, infertility, fertilisation, oocyte, pregnancy outcome and live births. Subsequently, a manual search of the reference lists of existing reviews and studies was also carried out against the inclusion criteria. After completing the scoping search, all titles were screened and abstracts retrieved against the inclusion criteria, which included original papers comparing the association between reproductive outcomes of patients who underwent IVF or ICSI treatment with ovarian endometriomas with no previous surgical treatment before IVF/ICSI and control participants. Control participants consisted of women who had not undergone previous ovarian surgery and had no history of endometriosis.

### Exclusion criteria

Studies focussing on women who had received medical or surgical treatment for their ovarian endometrioma before the IVF–ICSI cycle were excluded from the analysis. Single arm studies such as comparisons between ovaries affected with endometrioma and the contra-lateral normal ovary were also excluded. Literature reviews, non-original papers, duplication of a previous publication and non-English texts were also excluded.

### Data extraction and assessment of publication bias

**Fig. 1:**
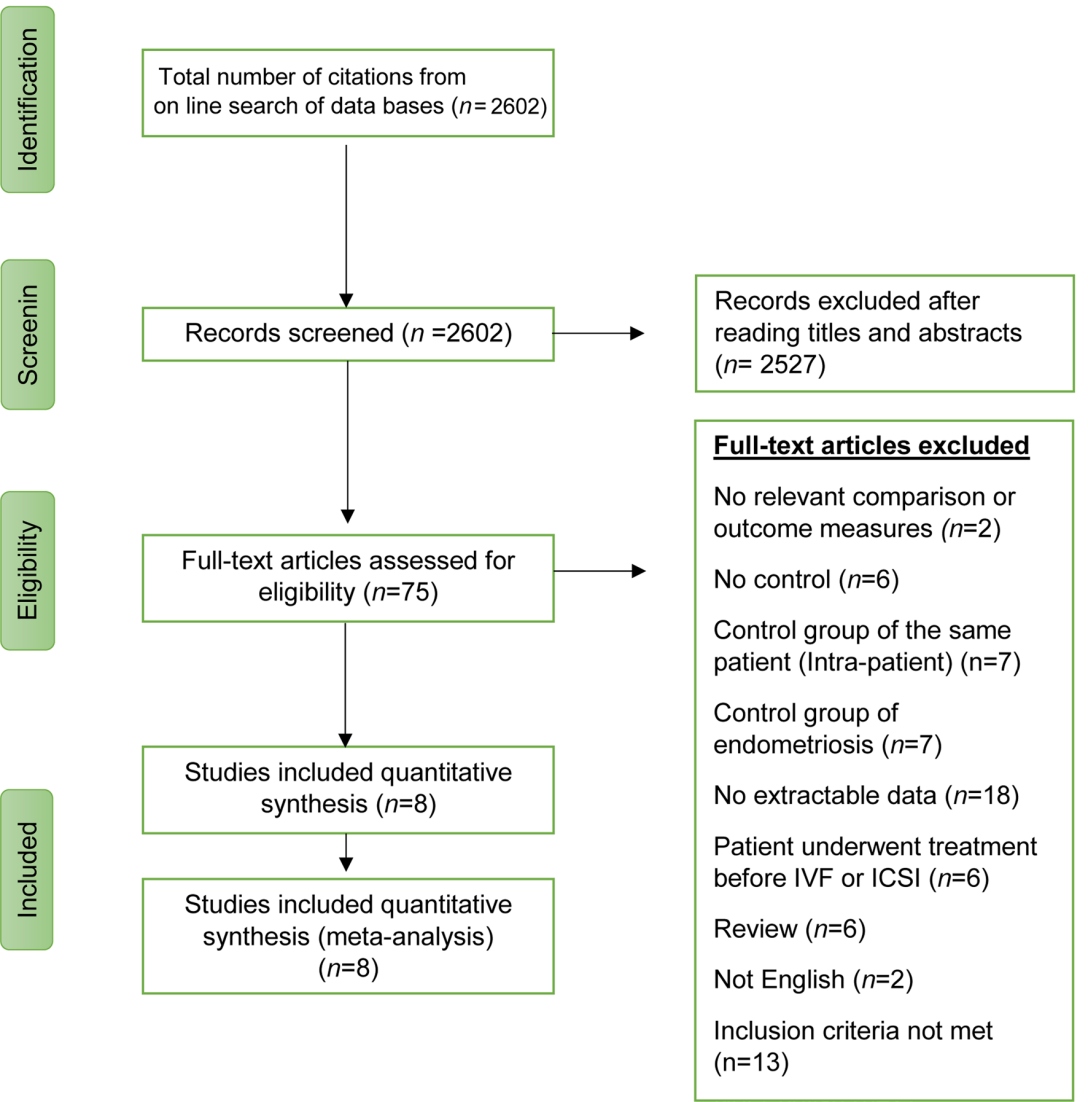
PRISMA chart of the literature search

Full manuscripts of all potentially eligible studies were assessed by two reviewers (SA and BN) for compliance with the inclusion and exclusion criteria. In case of disagreements regarding study eligibility, both authors arbitrated a consensus through a third reviewer (MM). The study selection method is illustrated in the PRISMA flowchart (Fig. 1).

For data extraction, a study characteristic table was constructed (Table 1). All relevant outcomes reported in the studies were collected, including duration of hormone stimulation, total number of oocytes retrieved, number of metaphase II oocytes retrieved, number of formed embryos and top-quality embryos, fertilisation rate, implantation rate, clinical pregnancy rate and the live birth rate wherever available.

**Table 1:** Characteristics of all studies included in the systematic

No	Study	Location	Study design	Duration	Intervention/protocol	Study group	*N*	Control group	*N*	Outcomes
1	Ashrafi [21]	Royan institute, Tehran Iran	Prospective cohort	2005–2007	Women undergoing ICSI	Women with either unilateral or bilateral unoperated ovarian endometrial cysts of less than 3 cm	(*n* = 47)	Women without ovarian endometriomas whose partner has mild male factor infertility	(*n* = 57)	Number of MII oocytes retrieved Number of embryos Number of top-quality embryos Clinical pregnancy rate Fertilisation rate Implantation rate Total dose of gonadotrophin (IU) Follicle number
2	Benaglia [30]	The infertility unit of the fondazione Ca’Granda Milan Italy	Retrospective cohort	2006–2010	Women undergoing IVF	Women with unoperated bilateral endometriomas	(*n* = 39)	Patients without endometriotic or non-endometriotic ovarian cysts	(*n* = 78)	Number of oocytes retrieved Number of embryos Total dose of gonadotropin Number of high-quality embryos Number of days of stimulation Implantation rate Clinical pregnancy rate Live birth rate
3	Bongioanni [32]	Three IVF units in Italy	Retrospective cohort	2004–2009	Women undergoing IVF	Women with unoperated endometrioma (≤ 6 cm)	(*n* = 142)	Women with tubal factor and without ovarian endometriomas	(*n* = 174)	Number of retrieved oocytes Fertilisation rate Implantation rate Total dose of gonadotropin Cancellation rate Pregnancy rate Live birth rate MII oocytes
4	Orazov [35]	The department of obstetrics and gynaecology with course of perinatology of medical institute of the RUDN university (peoples friendship university of Russia) and in the centre of reproduction and genetics ‘NOVA CLINIC’ Moscow Russian	Retrospective cohort	2018–2019	Women undergoing IVF/ICSI	Women recurrent intact unilateral endometriomas	(*n* = 70)	Women with tubal factor infertility	(*n *= 50)	Number of retrieved oocytes Number of days of stimulation Implantation rate Number of high-quality transferred embryos Number of embryos Anti-mullerian hormone Duration of ovulation induction
5	Ozgur [31]	The Antalya IVF clinic, Antalya, Turkey	Retrospective cohort	2014–2016	Women undergoing segmented IVF	Women with either unilateral or bilateral unoperated ovarian endometrial cysts	(*n* = 30)	Women without endometriomas	(*n* = 60)	Number of oocytes retrieved Number of MII oocytes retrieved Number of high-quality embryos Number of high-quality transferred embryos Antral follicle count (AFCs) Stimulation duration Fertilisation rate Implantation rate Ongoing pregnancy
6	Rakhimberdievicha [19]	The department of obstetrics and gynaecology with course of perinatology of medical institute of the RUDN university (peoples friendship university of Russia) and in the centre of reproduction and genetics ‘NOVA CLINIC’ Moscow Russian	Prospective cohort	2018–2019	Women undergoing IVF/ICSI	Women recurrent intact unilateral endometriomas,	(*n* = 50)	Women with tubal factor infertility	(*n* = 30)	Number of oocytes retrieved Number of MII oocytes retrieved AFCs Immature MI oocytes
7	Reinblatt [34]	The McGill reproductive centre, Montreal, Canada	Retrospective cohort	2006–2010	Women undergoing IVF	Women with unoperated bilateral endometriomas	(*n* = 13)	Women with male or tubal factor infertility without endometriomas	(*n* = 39)	Number of oocytes collected Number of MII oocytes retrieved Number of high-quality transferred embryos Number of embryos Cleavage rate Fertilisation rate
8	Yanushpolsky [20]	Harvard medical school Boston USA	Retrospective cohort	1994–1995	Women undergoing IVF	Women with intact endometrioma	*(n* = 37)	Women without any ovarian endometriomas	(*n* = 56)	Number of retrieved oocytes Number of days of stimulation Implantation rate Clinical pregnancy rate Live birth rate

The quality of each study was assessed using the Newcastle–Ottawa scale, in accordance with the MOOSE criteria and based on the recommendation of the Cochrane Collaboration for observational studies [25] (https://www.ohri.ca/programs/clinical_epidemiology/oxford.asp) (Table 2).

**Table 2:** Appraisal of methodological quality Newcastle–Ottawa Scale (NOS) assessment of the included studies in this meta-analysis

Study	Case-cohort representative	Selection of non-exposed control	Ascertainment of exposure	Outcome negative at start	Comparability by design^a^	Comparability by analysis	Outcome assessment	Duration of follow-up	Score
Ashrafi [21]	☆	☆	☆	☆	☆☆	☆	☆	☆	9
Bengalia [30]	☆	☆	☆	☆	☆☆	☆	☆	☆	9
Bongioanni [32]	☆	☆	☆	☆	☆☆	☆	☆	☆	9
Orazov [35]	☆	☆	☆	☆	☆	☆	☆	☆	8
Ozgur [31]	☆	☆	☆	☆	☆	☆	☆	☆	8
Rakhimberdievicha [19]	☆	☆	☆	☆	☆	☆	☆	☆	8
Reinblatt [34]	☆	☆	☆	☆	☆☆	☆	☆	☆	9
Yanushpolsky [20]	☆	☆	☆	☆	☆	☆	☆	☆	8

^☆^Indicates feature is available in the study

^a^ For comparability by design the checklist awarded a maximum of two stars (^☆☆^)

### Statistical analysis

Data analyses were conducted using Review Manager (RevMan) Version 5.3. (Copenhagen: The Nordic Cochrane Centre). Dichotomous outcome data were reported as odds ratios with 95% confidence intervals (CI) by applying the Mantel–Haenszel method to evaluate the pooled risk ratio with 95% confidence intervals [26]. Continuous data was synthesised using weighted means difference (WMD) with 95% CI. Heterogeneity was assessed graphically using forest plots and was statistically determined using the *I*^*2*^ statistic, which calculates the percentage difference between studies due to heterogeneity instead of sampling error [27]. An *I*^*2*^ ≥ 50% was considered to indicate substantial heterogeneity between studies. Scores below 50% were considered to represent low or moderate heterogeneity [28]. A random-effects model was applied in cases of high heterogeneity, and a fixed effects model in cases of low heterogeneity. A funnel plot was used to evaluate publication bias [29]

## Results

### Characteristics and quality of the included studies

The initial database search yielded a total of 2602 studies (Fig. 1). Articles with titles not related to endometriosis, endometrioma and reproductive outcomes, as well as review articles were not considered further. The remaining abstracts were reviewed and 75 studies deemed relevant for further investigation were identified. From this group, eight eligible studies were observational, reporting on a population of over 999 women. The characteristics of the eight studies included in the systematic review are shown in Table 1, and their quality as per the Newcastle–Ottawa Quality Assessment Scale is displayed in Table 2.

### Effects of endometrioma on reproductive outcomes

#### (1) Live birth rate

**Fig. 2:**
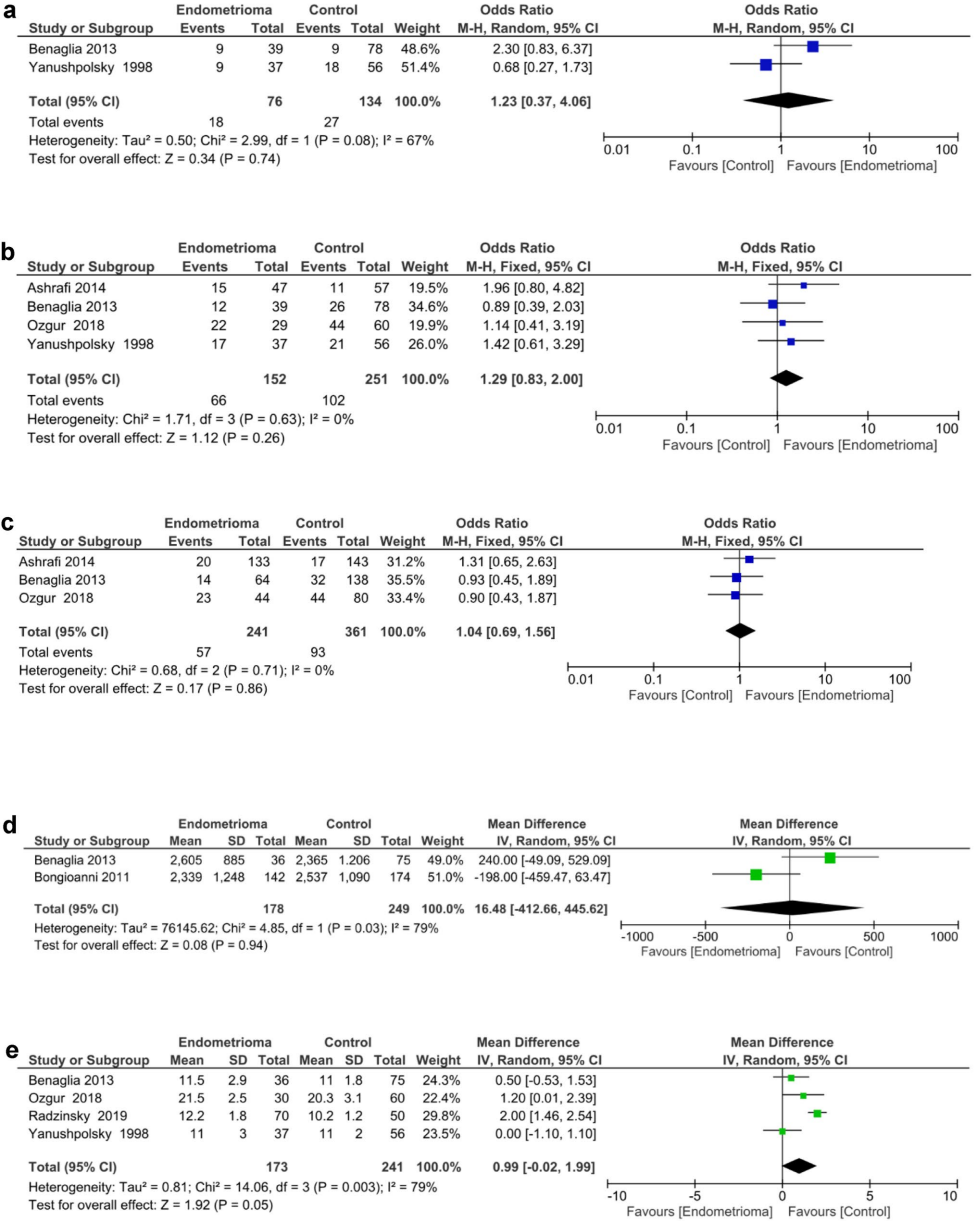

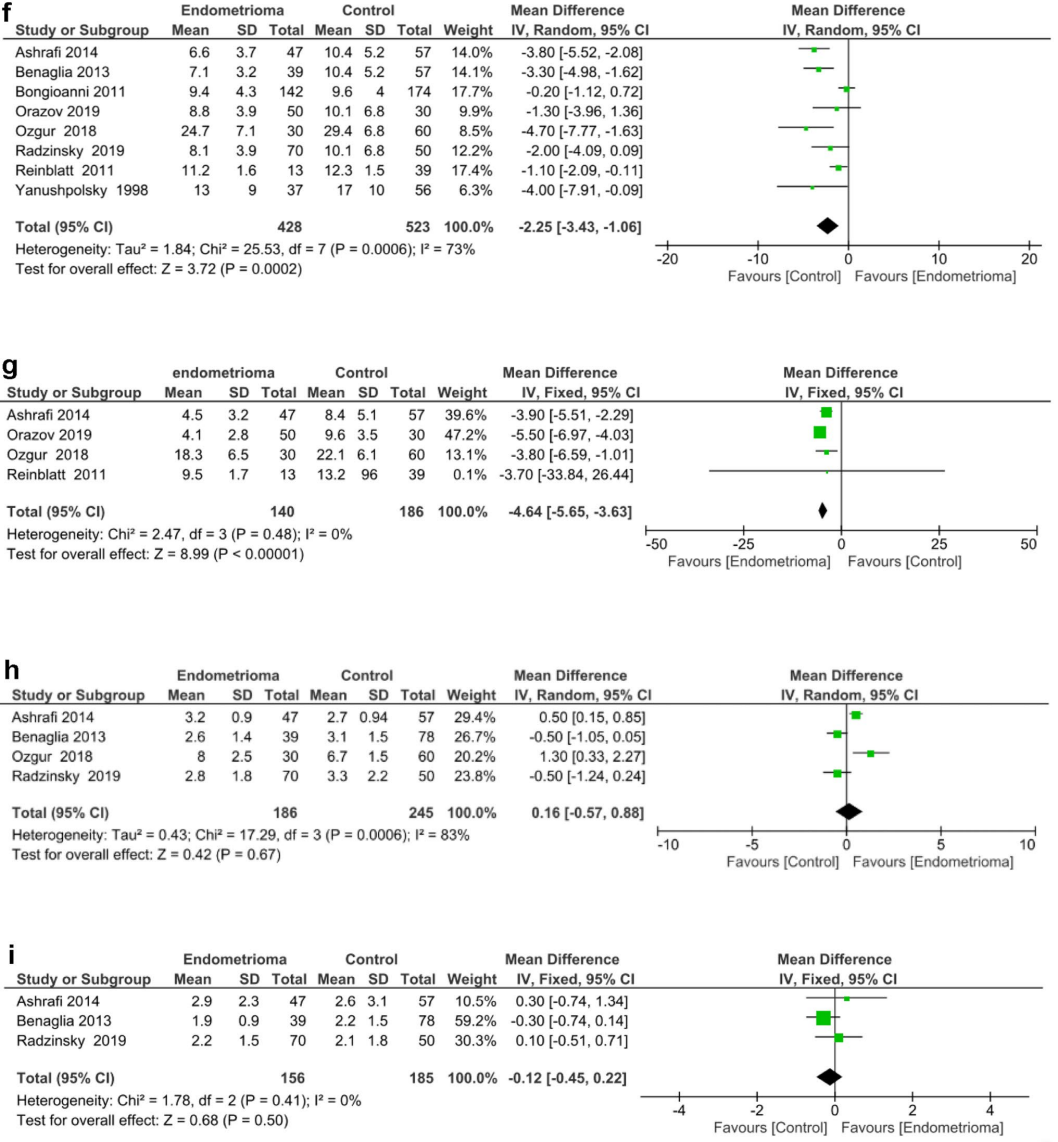
**a** Forest plot reporting the odds ratio (OR) between the endometrioma and control for live birth rate. **b** Forest plot reporting the OR between the endometrioma and control for clinical pregnancy rate. **c** Forest plot reporting the OR between the endometrioma and control for implantation rate. **d** Forest plot reporting the weighted mean difference (WMD) between endometrioma and control for the total amount of gonadotropin consumption. **e** Forest plot reporting the WMD between the endometrioma and control for the duration of gonadotropin stimulation. **f** Forest plot reporting the WMD between the endometrioma and control in the number of oocytes retrieved. **g** Forest plot reporting the WMD between the endometrioma and control for the number of MII oocytes. **h** Forest plot reporting the WMD between the endometrioma and control for the number of embryos. **i** Forest plot reporting the WMD between endometrioma and control for the number of high-quality embryos

Only two studies [20, 30] reported on the live birth rate. Pooled analysis revealed no significant difference in live birth rate between the endometrioma and control groups [odds ratio (OR) 1.23; 95% CI 0.37, 4.06] (*p* = 0.74). Significant heterogeneity existed among the studies as evidenced by an *I*^*2*^ value of 67%. Forest plots displaying the results of the meta-analysis for the live birth rate are shown in Fig. 2a.

#### (2) Clinical pregnancy rate

Four studies [20, 21, 30, 31] reported on clinical pregnancy rate. When this data was pooled together, no difference in clinical pregnancy rate was identified between the endometrioma and control groups (OR 1.29, 95% CI 0.83–2.0) (*p* = 0.26). No significant heterogeneity was found between the studies as shown by an *I*^*2*^ value of 0% (Fig. 2b).

#### (3) Implantation rate

Six studies [19–21, 30–32] reported on implantation rate. However, three of these studies were excluded as the data was in a non-usable format, so only the remaining three were analysed [21, 31, 33]. The implantation rate did not differ significantly between the endometrioma and the control groups when data from the three studies were combined (OR 1.04, 95% CI 0.69–1.56) (*p* = 0.86). Low heterogeneity was found to exist among the studies as shown by an *I*^*2*^ value of 0% (Fig. 2c).

#### (4) Total amount of gonadotrophin consumption

Two studies [30, 32] reported on the total amount of gonadotrophin administered (Fig. 2d). No significant difference was found between the endometrioma and the control groups [weighted mean difference (WMD) 16.48 international unit (IU); 95% CI 412.66–445.62] (*p* = 0.94). Results also showed significant heterogeneity between studies as shown by an *I*^*2*^ value of 79% (*p* = 0.03).

#### (5) Duration of gonadotrophin stimulation

Four studies [19, 20, 30, 31] reported on duration of gonadotrophin stimulation (Fig. 2e). No significant difference in the total duration of gonadotrophin stimulation was found between the endometrioma and the control groups. (WMD 0.99 days; 95% CI 0.02 to 1.99) (*p* = 0.05). Significant heterogeneity was found across studies as indicated by an *I*^*2*^ value of 79% (*p* = 0.003).

#### (6) Number of oocytes retrieved

**Fig. 3:**
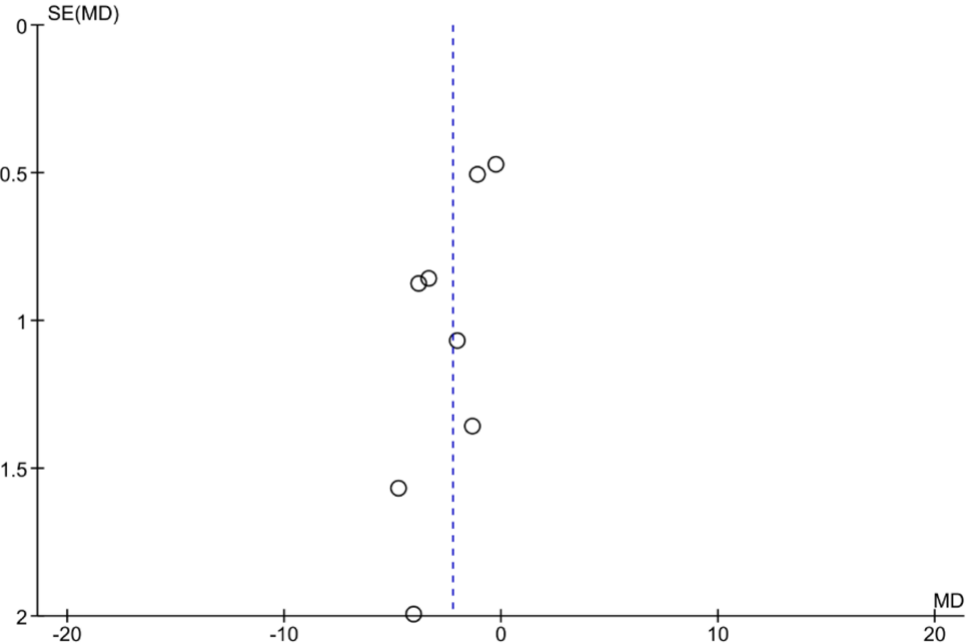
Funnel plot for studies comparing the mean number of oocytes retrieved from endometrioma patients versus control

All eight studies [19–21, 30–32, 34, 35] reported on the number of oocytes retrieved, which allowed quantitative pooled analysis. A significantly lower number of oocytes was retrieved from the endometrioma group relative to the control group (WMD-2.25; 95% CI 3.43 to − 1.06, *p* = 0.0002). Significant heterogeneity was found across studies as indicated by an *I*^*2*^ value of 73% (*p* = 0.0006) (Fig. 2f). The funnel plot was rather symmetric (Fig. 3), indicating no evidence of publication bias.

#### (7) Number of MII oocytes

Four studies [19, 21, 31, 34] reported on the number of MII oocytes retrieved. A significantly lower number of MII oocytes were collected from the endometrioma group compared to those in the control group (WMD -4.64; 95% CI 5.65 to − 3.63, *p* < 0.00001). The *I*^*2*^ value was 0%, showing low heterogeneity across the included studies (Fig. 2g).

#### (9) Number of embryos

Four studies [19, 21, 30, 31] reported on the total number of embryos. When these studies were considered together, no difference in the total number of embryos was detected between the endometrioma and the control groups (WMD 0.16; 95% CI 0.57–0.88) (*p* = 0.67). Significant heterogeneity was seen across the four studies as indicated by an *I*^*2*^ value of 83% (*p* = 0.0006) (Fig. 2h).

#### (9) Number of high-quality embryos

Three studies [19, 21, 30] assessed the number of high-quality embryos. Pooled results indicated that there was no significant difference in the number of high-quality embryos among the endometrioma versus the control groups (WMD − 0.12; 95% CI 0.45 to 0.22) (*p* = 0.50).There was no evidence of significant heterogeneity among the studies as shown by an *I*^*2*^ value of 0% (Fig. 2i).

## Discussion

This systematic review and meta-analysis explored the effect of ovarian endometrioma on reproductive outcomes in women undergoing IVF/ICSI treatment who had not been previously operated on. Compared to previous meta-analyses addressing this question, we purposely did not include any studies reporting on ART reproductive outcomes after surgical management of ovarian endometrioma. The rationale for this decision was that surgery could potentially compromise ovarian reserve and response to ovarian stimulation, thus behaving as a confounding factor [36, 37]. Furthermore, we excluded any single-arm study in which each patient was an index case and control to rule out any indirect systemic effects of endometriosis [38, 39].

Firstly, our review showed that while the presence of ovarian endometrioma can significantly reduce the number of oocytes and MII oocytes retrieved in women undergoing IVF/ICSI, it does not seem to adversely impact on the total amount of gonadotrophin administered, the duration of stimulation, the number of total and top-quality embryos, the implantation rate, clinical pregnancy rate and live birth rate. These results are supported by previous studies published by Ashrafi and Yang [21] [23] which showed that the presence of endometrioma negatively correlated with the number of oocytes retrieved from women undergoing IVF/ICSI when compared to controls. It is also important to note that endometriomas can act as a physical barrier that may hinder access to the ovary, consequently decreasing the number of the oocytes that can be retrieved [40]. Overall, this seems to suggest that the detrimental influence of endometriomas on ovarian function [12–17] does not seem to influence fertility outcomes in the context of assisted conception, once an embryo is fertilised.

Many studies have attempted to elucidate the mechanisms by which an endometrioma hinders ovarian function. Some studies argue that endometriomas might be detrimental to fertility by directly distorting the ovarian histology. Schubert [41] showed that follicle density is reduced in the cortex surrounding endometrioma when compared to other types of cysts. Maneschi [42] also reported on a decreased number of follicles in histological sections of the ovarian cortex surrounding the endometrioma, and proposed that the endometrioma may per se damage the ovary. Some studies suggested that the increase in size of the endometriomas could negatively adverse the ovarian reserve [43, 44], while others have shown size to have no effect [45, 46]. The discrepancy between studies may be related to the effect that size may have on the decision to surgically interfere, with a lower threshold to operate on larger endometriomas and a consequent loss of ovarian reserve. Further research in this area is therefore needed. Ovarian damage may be a result of oxidative stress [47–50], as the production of reactive oxygen species (ROS) affecting the ovarian cortex in the proximity of an endometrioma has been shown to be higher in comparison with other kinds of cysts [14]. Increased ROS production in the follicular fluid has been shown to have a significant negative impact on ovarian function [15]. These results are supported by a significant negative correlation between the increase in ROS production and reproductive outcomes, including oocyte quality, fertilisation rate, and embryo quality [49, 50]. Other changes in the follicular [51–53] and peritoneal microenvironment [54] may also have a negative effect on oocyte numbers and quality by affecting oocyte metabolism and DNA integrity [55, 56].

At the present time, the generally accepted idea is that endometriomas might induce a quantitative, but not a qualitative damage to the ovarian reserve [16, 57]. In other words, even if the collected number of oocytes is reduced, pregnancy outcome is not altered. Pathogenic mechanisms causing this damage have not yet been fully elucidated.

This review showed that there a lower number of MII oocytes retrieved from women with endometriomas which is an agreements with other studies [23, 35]. However, some studies showed no negative effect of an endometrioma on MII oocytes [34, 58]. It is worth mentioning that many of these previous studies were small and not adequately powered, and hence prone to type 2 error; moreover, some lacked a control group [34, 58]. Another possible reason for the discrepancy between our review and some of the published literature is that the current oocyte morphology scoring systems used to assess intrinsic egg quality are rather subjective and prone to high inter-variability [59, 60]. As a result, predicting embryo quality can be challenging and biased. Further prospective clinical studies with adequately powered sample sizes that correlate clinical outcomes with molecular and cellular findings are needed to better understand the pathogenic effect of endometrioma on ovarian function. Many studies indicate that endometriosis affects oocyte morphologic and molecular characterisation. Goud [61] conducted functional studies assessing MII oocytes collected from endometriosis patients compared to women without endometriosis. They determined that oocytes from endometriosis patients showed increased cortical granule loss and zona pellucida (ZP) hardening, which could affect the ability of the embryo to undergo hatching and implantation [62].

Secondly, our study demonstrates that once fertilisation has occurred, the presence of an endometrioma does not seem to affect the number of total (and high-quality) embryos, which is consistent with previous studies [16, 23]. Despite the fact that our paper did not look at the frozen embryo cycle, evidence from the literature suggests that cumulative pregnancy rates from fresh and frozen cycles are not affected by the presence of endometriomas [63]. Robust data regarding embryo development is lacking and therefore, it was not formally addressed in the current review. Future studies may benefit from a comparative examination of women with unilateral endometriomas in order to examine embryo development in the affected versus the normal ovary. Furthermore, the use of an objective assessment method such as time-lapse technology may be useful to optimise morphological assessment of embryo quality and mitigate variations across different embryo grading systems.

Thirdly, our review did not show significant differences in the requirement of gonadotrophin between women with and without endometriomas. These findings are in agreement with a previous study by Yang [23], but oppose what was reported by Al-Azemi [64]. We hypothesised that the relatively poor response to gonadotrophins reported by the Al-Azemi [64] in the endometrioma group could be a consequence of the deleterious effect of surgery on the endometriomas. This untoward effect is not reflected in our study as we purposely excluded women who had received surgical management of their endometriomas. Although the study by González-Foruria*.*[65] showed that the Ovarian Sensitivity Index (OSI) in endometriomas group was significantly lower compared to the control group (3.3 ± 3.8 versus 5.1 ± 8.2; *p* < 0.001), in our study, although we did not use that index, we found no difference in the amount of gonadotropins for ovarian stimulation between the two groups.

Finally, the findings of this study demonstrate that endometriomas did not have a significant effect on the implantation rate. This suggests that endometrial receptivity is not affected in the presence of endometrioma [66]. Most importantly, the clinical pregnancy and live birth rates were similar between the patients with and without ovarian endometrioma.

The findings of our study demonstrate that the mere presence of endometriomas does not hinder fertility chances. These findings as well as the findings of other studies therefore do not support the excision of endometriomas, due to the potential detrimental effect of surgery on the ovarian reserve [46, 67]. Accordingly, there is an increasing body of evidence that endometriomas should only be removed if they are associated with pain or if their presence will significantly impede access to the ovary during oocyte recovery.

The search strategy employed in the present meta-analysis was broad, and the quality of the included studies was considered high. However, several outcomes showed significant heterogeneity across studies. This heterogeneity can be accounted for by differences in the unilaterality/bilaterality of the endometriomas, the size of the endometrioma and the general extent of pathology. Some studies have shown that small single endometriomas do not appear to affect ovarian function in the context of ART [68]. In an attempt to minimise this variation, a random-effects model was applied for combined outcomes in cases of high heterogeneity such as in case of LBR and oocyte numbers but a subgroup or sensitivity analysis was not possible due to the limited data and sample size.

## Conclusion

Women with endometriomas undergoing assisted conception procedures seem to have a lower mean number of oocytes and MII oocytes retrieved when compared to those without which suggests that the presence of the endometrioma (and its underlying disease) can have a detrimental effect on ovarian function. However, this study did not find any significant difference in gonadotrophin requirements, total number and quality of embryos, implantation rate and pregnancy live birth between the two groups. However, given the lack of clinical studies examining the effect of endometrioma on embryo quality as highlighted by our review, we believe that additional randomised controlled trials with adequately-powered sample sizes will be crucial to further validate our findings.
